# Supple bodies, healthy minds: yoga, psychedelics and American mental health

**DOI:** 10.1136/medhum-2017-011422

**Published:** 2018-03-30

**Authors:** Lucas Richert, Matthew DeCloedt

**Affiliations:** 1 Faculty of Humanities and Social Sciences, University of Strathclyde, Glasgow, UK; 2 Central European University, Budapest, Hungary

**Keywords:** psychiatry, psychology, arts In health/arts and health

## Abstract

Much discussion about mental health has revolved around treatment models. As interdisciplinary scholarship has shown, mental health knowledge, far from being a neutral product detached from the society that generated it, was shaped by politics, economics and culture. By drawing on case studies of yoga, religion and fitness, this article will examine the ways in which mental health practices—sometimes scientific, sometimes spiritual—have been conceived, debated and applied by researchers and the public. More specifically, it will interrogate the relationship between yoga, psychedelics, South Asian and Eastern religion (as understood and practiced in the USA) and mental health.

During the 1960s countercultural movement, many Americans saw yoga and psychedelic drugs as equally valid routes to spiritual fulfilment and mental health. As the *San Franciso Oracle* put it in 1965: ‘Yoga and psychedelics. Different means, same end’ (Syman, p. 218–219).^1^ Researchers have since uncovered ‘significant evidence’ that yoga and other practices ‘may induce specific classes of ASC (altered states of consciousness)’.^2^ ^i^ And some ‘drug-induced experiences bear striking resemblances to mystical experiences’, with drug-takers reporting, to varying degrees, a ‘greater appreciation and understanding of Asian philosophies’ post-trip.^3^ Whatever the pathway, spiritual seekers in the 1960s USA likely encountered both yoga and psychedelics, expanding their minds while toning their muscles. This article will explore the reasons why some Americans, many of whom were situated in universities as well as based on the East and West coasts, wanted to escape, how they did so and the legacy of these two practices. It captures some of the key figures who shaped ideas about yoga, psychedelic drugs, Eastern religion and the mental health field.

Americans were receptive to novel thinking and practices in the 1960s. Ecologism, conservationism, vegetarianism, veganism and yoga all came to the fore (Sessa, p. 107).^4^ Messages of the counterculture pushed notions of personal responsibility for health, corporeal and spiritual, and led many Americans to strive for perfection. With society in flux, people sought peace of mind. As the psychologist Abraham Maslow put it, Americans were adrift in a postmodern world: “We’re stuck now in our own culture…stuck in a silly world which makes all sorts of unnecessary problems”.^5^ Mental health, by many accounts, suffered as a result. Thus, transcending the present was one means of coping. Eastern religion, experimentation with yoga and psychedelics seemed a useful alternative to American capitalism, conservatism, militarism, racism, violence and the prospect of nuclear war. Not only that, the experimentation seemed a useful alternative to other mental health strategies (Sessa, p. 99; Sacks, p. 90ff; Osto, p. 41–207).^4 6–8^ ^ii^ Yogis and drug takers were after the same thing: an experience that reframed the way they looked at the world and themselves. Whether they saw their actions as a form of therapy to treat mental health issues or religious exercise was beside the point. Semantics were less important than the feeling they were chasing. Americans surveyed foreign cultures and religions, and borrowed and adapted as they pleased. As such, the 1960s was an era in which syncretism and cult-like organisations flourished.^9^ America, the cultural melting pot, had no reservations about taking what it liked from the East and adapting it to the West (Osto, p. 78).^8^ Form was not as important as content. Several thinkers were crucial in influencing the combination of psychedelics and yoga. To them, supple bodies were related to healthy minds.

Contemporarily, positive accounts of mental health services gave way to increasingly critical approaches. ‘American psychiatry seems to be in a state of confusion’, was what many people felt, and psychiatrists themselves had become ever more ‘sceptical’.^10^ ^iii^ This paper will add to the discussions about mental health in the 1960s through the lens of yoga and psychedelics. The following pages will build on recent scholarship about yoga practitioners, the role of medical humanities in mental health and current examinations of the life of breath.^11 12^ ^iv^ If the medical humanities are open to ‘considering alternative perspectives on the multiple materialities of the world beyond its textual inscriptions’, as Philo *et al* have pondered, then this article should be regarded as a contribution to alternative health.^13^ Using archival materials, as well as medical and popular literature, this work will explore yoga as a health movement, a tool of mental health promotion, and tackle its commercialisation.

## Yoga as an American fitness trend

Yoga captivated certain Americans as far back as the middle of the 19th century. Henry David Thoreau famously called himself a yogi in 1849. Others were intrigued by the supernatural possibilities of ‘human hibernation’, levitation or simply the opportunity of enhanced sexual powers. Yoga was a niche pursuit at this time, to put it mildly. In 1893, Swami Vivekananda, a monk from Calcutta, advertised India and Hinduism at the Parliament of the World’s Religions in Chicago. At the same time, he introduced yoga to a greater number of Americans. He subsequently toured the USA delivering lectures promoting his book *Raja Yoga.* 14–16 Worried Americans would ‘abuse certain elements of the practice and its philosophy’, Vivekananda repackaged yoga to prevent them from, as he saw it, corrupting the tradition (Syman, p. 8).^1^ Yoga soon spread throughout the West at the turn of the 20th century. According to Mark Singleton, yoga’s popularity increased because it aligned with other health and fitness trends of the time, especially Nordic gymnastic drills.^16^ This new form of yoga was wholly demystified.

By the early 1920s, Pierre Bernard, known as ‘The Omnipotent Oom’, and Blanche DeVries opened the Clarkstown Country Club in Nyack, New York, for the upper crust of the Northeast. Yoga constituted part of a programme that focused on ‘health and well-being rather than…religious practice’.^17 18^ It was a form of elitist, conspicuous and worldly consumption. In 1931, Jagannath G Gune published *Asanas*, a book that showcased yoga postures for a wider audience. However, it was Swami Sivananda, an Indian yogi, who truly commercialised yoga in the West through his English-language publications and Divine Life Society in the 1930s.^19^ Yoga, or at least a modified version of it, was gaining momentum in America.^20^

Scientists and medical practitioners took an interest in the exotic practice too. Sivananda drew the attention of academics like Dr Kovoor T Behanan of Yale University, who spent a year in India at Gune’s ashram studying the practice before returning to New Haven to assess his findings.^21^ Yogic breathing and its ability ‘to arouse little by little…this indomitable power of the human mind’ fascinated Behana.^22^ Others, like the Chicago-based physician Edmund Jacobson, were concerned with yoga’s use in the clinical treatment of headaches and depression. Carl Jung is one example of a mental health professional who assessed yoga’s value *and* its dangers.^23^ In 1932, he emphasised the link between yoga, sex and a ‘deliberately induced psychotic state’.^24^ Still, yoga was most associated with fitness in the early 20th century, with the science behind it largely ignored.^25^

In the late 1940s and early 1950s, yoga studios opened shop across the USA, from Hollywood to New York. The marketplace began to offer multiple varieties to cater to American consumers, offering up choices that addressed both mind and body. Theos Bernard’s *Hatha Yoga: The Report of a Personal Experience* was published in 1947, which served as a ‘major sourcebook’ for yogis in the 1950s.^26^ Indian gurus, too, began attracting large followings, promoting a yoga mostly devoid of a religious or intellectual foundation.^27^ Free-spirited groups led by charismatic gurus dominated the yoga scene in the 1960s, but they were soon competing with a more commercial brand of their art. Yoga appeared on American television in 1961, when Richard Hittleman brought ‘non-religious yoga…with an emphasis on its physical benefits’ into America’s living rooms. Yoga was gradually becoming mainstream, and by the 1970s it had saturated American culture.^26^ While most practiced yoga for the body, a few devotees began to concentrate on its mental effects.

Interest in the spiritual aspects of yoga, and Eastern religion in general, should not be understated. It tacked closely to the 1950s–1960s psychedelic revolution (Osto, p. 55).^8^ Drug-takers, posited psychologist Bernard Aaronson and psychiatrist Humphry Osmond (coiner of the term ‘psychedelic’), recognised ‘that each individual consciousness is located in the body’ and people turned to yoga to strengthen their spiritual muscles. Correctly done, individuals could attain ‘peace, co-ordination and good bodily functioning’, ‘increased awareness of the body’ and altered states of consciousness (Aaronson, p. 26 and p. 276).^28^ This took hard graft, however. American yogis and yoga students did not always want to invest the requisite effort to achieve altered states of consciousness and lucidity through either meditation or artful poses. The idea that one had to sacrifice—to work hard—to achieve transcendence was difficult for ‘impertinent, scientifically minded and intellectually curious Westerner[s]’ to accept (Aaronson, p. 132).^28^ The following section of this article examines more closely the relationship between psychedelics, yoga and altered states of consciousness.

## Mental health and syncretism

Yoga and psychedelics flourished against the backdrop of fluctuating mental health practices and 1968 was certainly an important year. It ‘rocked the world’, ‘changed everything’ and ‘made us who we are’.^29 30^ The new head of the American Psychiatric Association (APA), Raymond Waggoner, was in touch with the moment. He claimed he would oversee ‘healthy and wisely determined progress…’^31^ Psychiatrists, he argued, ought to take a more active role in social problems outside the field. It was a professional environment shaped by such psychiatrists as RD Laing, David Cooper, Joseph Berke and Leon Redler, who produced early critiques of psychiatry. Franz Fanon, the French psychiatrist and philosopher, also published the eighth edition of *The Wretched of the Earth* in 1968, while Thomas Szasz, another critic, published *Law, Liberty, and Psychiatry* that same year. Socially conscious psychiatrists in the APA coalesced during the annual convention in Boston and underlined issues of war, race, class and gender. They voiced concern about how breakthroughs in military hardware, advancements in human rights activism and the rising automation of Western civilisation affected Americans’ mental health.^32–34^

Drugs were a vital part of the era. In 1968, a series of articles in mainstream psychiatry debated the dangers of cannabis, linking it to psychosis.^35–42^ The *American Journal of Psychiatry* and *Psychiatric News* disapproved of its use. The era of psychedelic medicine was drawing to a close. Authorities in the UK moved against LSD and in 1966 it was made illegal. Medical use also stopped and was prohibited by the Misuse of Drugs Act when it came into force in 1973. LSD was banned in the USA in 1968. *The British Journal of Psychiatry* (*BJP*), for its part, was engaging with the major debates of the time. The October 1968 issue evaluated LSD. *BJP*’s focus on this mind-expanding drug, among others, represented evolution in sociomedicine and a free marketplace of ideas in which yoga and psychedelics might flourish as an alternative.^43^

Psychedelics, between the 1940s and 1970s, seemed like the next frontier in psychiatry and self-awareness. Research and experimentation flourished. LSD, in particular, ‘spawned a global interest in the cross-cultural dimensions of hallucinogens’ during the 1950s (Sessa, p. 194).^4^ Many researchers working in the mental health field expected to produce a ‘wonder drug’ that would provide an answer for the ‘remaining questions about human nature’.^44^ But the scientific community was not solely responsible for the rise of psychedelics. Nor were drugs seen as the only path to well-being. Eastern-influenced thinkers across the West, and from a variety of disciplines, were vital in exploring altered states of consciousness for answers to society’s problems.

Mysticism constituted a central part of the psychedelic credo (Osto, p. 38).^8^ ^v^ In 1958, Aldous Huxley, the English author and exponent of psychedelic experimentation, argued that LSD ‘lowers the barrier between conscious and subconscious and permits the patient to look more deeply and understandingly into the recesses of his own mind. The deepening of self-knowledge takes place against a background of visionary and even mystical experience’. Self-exploration was a key component to patients maintaining their mental health. Although fleeting, these drug-induced experiences, or ‘spiritual exercises’, could be ‘enormously helpful’.^45^ Huxley addressed claims that his was a ‘mere chemical religion’ by pointing out the corresponding physiological and biochemical effects of yoga and other spiritual practices.^46^ Indeed, both religious and psychopharmacological methods, he stated, were ‘devices for altering the chemistry of the body…’ The ‘breathing techniques taught by the yogi of India’ changed the ‘concentration of carbon dioxide in the blood; and the psychological consequence of this is a change in the quality of consciousness’. The benefits of such changes, Huxley argued, would be manifold, because psychedelics would lead to a type of widespread religious revival based on ‘radical self-transcendence’ (Sessa, p. 62–33).^4^ ^vi^ He believed mind-altering substances had the capacity to change humankind’s mental state for the better. More generally, Huxley suggested that drug-induced religious experiences should at least be treated the same as their ancient mystical counterparts. For better or worse, intellectual engagements of this sort did not always preoccupy those looking for altered states of consciousness in the USA.

Another significant thinker behind the psychedelic credo was Abraham Maslow. A proponent of self-realisation, he agreed that psychedelics had potential, but worried they might cause harm to users’ mental health.^47^ Maslow was first drawn to Eastern thought in the 1960s realising that Americans were sick and in need of spiritual awakening. In a fragmented society, people were more alone than ever. He understood that the interest in the East reflected America’s desire for ‘altruism, compassion, aesthetics, courage and other traits’. Yet, the counterculture’s hijacking of the East bothered him. Instead of focusing on the transcendent by itself, through yoga or psychedelics, Maslow believed that confronting the quotidian led to self-actualisation. In his view, ‘to be looking elsewhere for miracles is a sure sign of ignorance that *everything* is miraculous’. He encouraged his patients to work towards ‘plateau experiences’, or extended periods of serenity, as opposed to transitory peak experiences. One had to expend energy for these states ‘through conscious, diligent effort’^48 49^ ^vii^ and psychedelic trips were a cheap reproduction of genuine mystical experiences.

Psychotherapeutic models advocated by Maslow (and others) negotiated with countercultural alternatives. From the 1960s, ‘travellers on the hippie trail ended up in Indian ashrams’ and generated enthusiasm for Indian religion and yoga.^16^ This ‘hip subculture’ combined various traditions with ‘the chemical mysticism of psychedelic drugs’. Why? Alan Watts, a religious philosopher and psychedelic aficionado in California, believed this blending to be a ‘responsible effort of young people to correct the soul-destroying course of industrial civilization’.^50^ He argued that such syncretic practices were within the ‘tradition of genuine religious involvement'.^51–53^ ‘The Western man’, wrote Watts, ‘has arrived, by chance…at a state of consciousness in which he experiences directly and vividly’ the unity of the universe. As he put it, psychedelics—although not without risk—were an avenue to tap into that singularity.^54^ Youth were fed up with the state of the world and, if they could not change it outwardly, wanted to transform it from within.

The heady mixture of psychedelics and yoga was also driven at the institutional and individual level. The famous Esalen Institute, near Big Sur, California, was founded in 1962 by Stanford graduates Michael Murphy and Richard Price. It encouraged the development of ‘human potential’ by offering clients Eastern-influenced spiritual lessons in yoga and other disciplines like gestalt, transactional analysis and reflexology (Osto, p. 259).^8 55–57^ Psychedelic-induced experimentation was officially forbidden, but with workshops led by propsychedelic psychologists like Timothy Leary and Richard Alpert, many attendees explored ‘altered states of consciousness’ regardless^58 59^ (Grogan, p. 176).^60^ Part of the 1960s psychological revolution, Esalen offered ‘a more humanistic and holistic view of the person’ that combined New-Age alternative treatment models with radical psychopharmacological drug treatment and ancient philosophy.^61^ Although not a cult, Esalen’s leaders followed paths towards enlightenment like their ancient forbearers Buddha and Jesus and wanted to spread what they learnt to their fellow Americans.

Murphy was inspired to found Esalen by Sri Aurobindo, an Indian philosopher and yogi. He had spent several months at the guru’s retreat in Pondicherry, where he absorbed Aurobindo’s cocktail of Eastern and Western thought that combined yoga and psychology. It was a perfect fit for what Murphy and Price wanted in California. These were ideas that would allow individuals to explore their human potential and spirituality. Yoga in particular, claimed Aurobindo, could ‘bring down a divine nature and a divine life into the mental, vital and physical nature and life of humanity’ (Grogan, p. 160–162).^60^ Richard Alpert (later Ram Dass), similarly, was able to take a transformative trip to India, where he immersed himself in Hinduism, Buddhism, meditation and yoga. He combined these perspectives with his psychedelic research, investigating the intersection of drugs with spirituality (Walsh, p. 222).^62^ Alpert reported, for example, that psychedelics gave greater spiritual and social meaning to sex and allowed sexual ‘partners (to) transcend the subject-object relationship…and merge into the unitive experience’. He added: ‘Students of sexual yoga already are familiar with this model’.^63^ Alpert viewed psychedelics, especially LSD, as the ‘sacraments’ of the countercultural movement and believed ‘that those religions which still provide an opportunity for direct religious experience will find a place for the psychedelic chemicals’. One could ‘trip’ towards God in the Downward Dog or on LSD.^64^ Alpert’s 1971 book, *Be Here Now*, reflected this view, which pooled all manner of religious traditions with yoga and psychedelics (Osto, p. 40).^8^ After all, these myriad paths had the same destination in mind.

For instance, Jack Kornfield, a clinical psychologist and Buddhist teacher, recalled his initial encounter with psychedelics after studying Eastern religion at Dartmouth College. ‘The majority of Western Buddhist teachers’, he noted, ‘used psychedelics at the start of their practice’ in the 1960s. This was because drugs served as a shortcut on the path to the Buddhist heights only attainable through yoga, prayer and meditation (Sessa, p. 91).^6^ By marrying psychedelics together with ancient religious practices, Kornfield believed ‘there might be some very fruitful’ results for spiritual life (Osto, p. 10–11).^8^ He and others in the field, however, worried that a dependence on psychedelics to achieve altered states of consciousness was problematic. Learning to achieve these states through practice was more sustainable over the long term. Psychospiritual transformations could thus be achieved in appropriate and inappropriate ways.^65^ Furthermore, there was a risk of ‘burn-out’ or the development of a mental health disorder.

What did spiritual-seekers experience? Who were they? And what was it they were looking for? Researchers highlighted several shared aspects of the ‘psychedelic experience’, as reported by drug-takers. These included unity with the subject’s self; insight into objectivity and reality; transcendence of space and time; a sense of sacredness; a deeply felt positive mood; an awareness of paradoxicality; the ineffability of describing mystical consciousness; the transience of subjectivity and experience and positive changes to attitude and behaviour. Yogis, interestingly, had also reported such phenomena (Sessa, p. 21–23, p. 66–67).^4 66–68^ ^viii^ Commonalities were not new, having been suggested by thinkers like Aldous Huxley in the late 1950s. Yet, shared ideas and experiences also buttressed the syncretic nature of yoga and drug-taking.

As for the identities of the spiritual-seekers, many came from privileged backgrounds and took full advantage of their college years to transform themselves from the inside out. Students in Cambridge, Massachusetts took seriously New-Age beliefs and practices. They began ‘with Asian philosophy’, and tried meditation, then yoga. ‘And underlying everything’, according to one witness, was psychedelic drugs. ‘They were *on* the drugs…they were meditating for hours on end…And they weren’t plebeians. Intellectually they were aristocrats…’ (Smith, p. 42).^69^ Anecdotes like this abound, and are backed up by hard evidence that the followers of ‘psychedelic Buddhism’ are ‘predominantly white, middle-class or upper-class, college educated and politically liberal’ (Osto, p. xi).^8^

To their proponents, however, psychedelics were as essential in their ‘spiritual seeking’ as yoga. Even Eastern-trained individuals noticed the symmetry of experience in religious practices and psychedelic experimentation and suggested the latter might be spiritually beneficial.^70^ Still, many wanted to put yoga ‘back in the temple, where they believed it belonged’ (Syman, p. 216).^1^ It more authentic and safer.

## Bad dharma: from highs to cultural hijacking

Pushback against syncretism emanated from multiple directions. A 1966 *Psychedelic Review* article, for example, clearly illustrated the dangers of the drug-taking pathway. John Blofeld, a yogi living in Bangkok, had read Aldous Huxley’s claim ‘that mescaline can induce yogic experiences of a high order’. While sceptical, Blofeld took the drug under supervision at his home and recorded his experiences in a phenomenological fashion. After a short period of pleasantness, he had ‘the sensation of a rapidly fragmenting personality’ and his ‘mental stress grew agonising’. Blofeld felt as though he was a ‘disembodied sufferer’ and that ‘Hell itself could hardly be more terrifying’ than his experience. Then the direction shifted. ‘From hellish torment’, he recounted, “I was plunged into ecstasy”. He felt the ‘undifferentiated unity’ of the world, ‘unutterable bliss’ and an awareness of ‘the Buddhist doctrine of dharmas’. He was confounded about how ‘a dose of mescaline can make such an experience possible to someone who has not yet attained it by the profound and prolonged practice of yogic meditation…’ As a yogi, he noted that a drug might produce a ‘mystical and perhaps quasi-religious’ altered state of consciousness.^71^ The possible mental anguish of a bad trip, Blofeld held, was worth the risk. When things went well, psychedelics provided one with a truly transformative experience.

While risk/reward tensions were present from the start, in the later 1960s several thinkers began to disavow the syncretic use of psychedelics entirely. The pushback to yoga and drugs centred on arguments about the cultural appropriation of Eastern spiritual language and metaphors. Yoga gurus with ‘large American followings’, writes Stefanie Syman, regularly ‘dismissed psychedelics…and frowned on any mingling of drugs and discipline’ (Syman, p. 225).^1^ Some gurus believed drugs ‘could cause “spiritual dryness”, “disbelief” and even permanent brain damage' (Syman, p. 203–208).^1^ Indian yogis had long valued altered states of consciousness, but some felt that drug-related experiences had the potential to be inconsequential and trivial as regards *dharma*. Worse still, they might be dangerous (Smith, p.144–145).^65^ Neophytes ought to be wary. Ecstasy (and not the MDMA kind) was not to be treated lightly. None of these cautionary warnings, though, prevented countercultural icons like Aldous Huxley, Timothy Leary and Richard Alpert from mixing psychedelics and spirituality. Huxley and others ‘had come to psychedelic drugs through their interest in yoga or Eastern religion’ in the first instance (Syman, p. 218).^1^ What was acceptable to outsiders, therefore, was borderline offensive to traditional teachers.

A notable example was the Psychedelic Venus Church in California, which built drugs (and explicit sexual practices), into all of its religious activities. Promotional materials called it a ‘pantheistic nature religion of hedonism’ and in an ironic message deemed ‘Official Dogmatic Bombast’, declared that ‘*Cannabis sativa* is the preferred sacrament of this church; although psychedelics (and even wine) may sometimes be used’.^72^ To achieve greater peace of mind and enlightenment, church rituals included blind meditation and touch, nude group sensitivity encounters, nude yoga, cannabis communions and OM chanting. The church’s manifesto was a tangle of positions and ideas. It outlined that members, including the founder, Jefferson Fuck Poland, did not ‘commit ourselves to one creedal formula of words’, that Venus-Aphrodite served as lead God and mental tranquillity would be achieved through sexual freedom. Social issues of the day were paid lip service; the manifesto noted that ‘we will do what we can to prevent warfare, racism, sexism and ecological disaster’, but ‘most of our activities will be on the level of personal liberation’.^73^

In *Nelly Heathen*, the major but short-lived periodical of the church, readers were exposed to erotic imagery, lessons about getting high and the value of yoga. Indeed, a section was devoted entirely to yoga. Versions of Ravi Kumar’s *Tantra Asanas* were printed, highlighting that ‘energy centres containing latent psychic powers’ could be unlocked with sex and yoga. Here, breathing techniques were emphasised. Other articles asked ‘Are the Hindu Gods destructive?’ and ‘Are we being Easternized?’ In a third article, ‘True Hedonism’, the author suggested that yoga was a panacea for mind and body, as well as a mode of pleasure. ‘Yoga provides two paths to True Enlightenment. The easier way involves discipline and denial. The other way, through pleasure, is considered much more difficult because it is so easy to get lost’. When combined with other Venus Psychedelic Church practices, with its leaders acting as guides, the article ended with a question: ‘what could be more pleasant than true hedonism’.^74^

Meher Baba, a Sufi spiritual teacher in India, who was highly critical of Leary, Blofeld, and groups like the Venus Church, served as a critic of the undermining of traditional practices. In 1966, he penned the antipsychedelic tract ‘God in a Pill?’, where he described LSD, mescaline and psilocybin-fuelled ‘spiritual’ experiences as ‘superficial’. He also described notions of consciousness expansion as illusory. Psychedelics were ‘positively *harmful*’ for the spirit, according to Baba, and he believed such substances should only be used ‘when prescribed by a professional medical practitioner in the case of serious mental disorder under his direct supervision’. LSD, for example, had many practical applications, but to reach the divine there was no synthetic shortcut. Mental health treatments were not, in Baba’s view, to be used for spiritual ends. Only through love of, and unity with, God can one attain spiritual progress, he espoused.^75 76^ Bruce Hoffman, an English instructor at Rockland Community College in Suffern, New York, recalled being told that Meher Baba said ‘If God can be found in a pill, he’s not worthy of being God’.^77^ But Baba’s own mysticism functioned as a kind of ‘*substitute gratification* for psychedelic trips’ and was extremely attractive to former drug users versed in Eastern religion. Baba’s poses, that is, acted as surrogate pills; both were designed to enhance ‘self-insight’ and ‘consciousness expansion’.^78^ To consumers of yoga, psychedelics and Eastern religion there remained little to distinguish between these practices.

After all, getting ‘highs’ from God, meditation and yoga was easier to integrate into daily life than ‘incapacitating…chemical highs’.^78^ Baba appealed to the psychedelic instinct of his followers when he promised ‘Baba trips’ by looking inward.^79^ But looking too far inward could be just as dangerous as drugs. Many Zen Buddhist teachers, for instance, warned their students of the ‘danger of possession by demons’ when in certain states of mind. It was best to avoid ‘temptations to go into a stupor, toy with paranormal psychic functions, indulge in ecstasies or quietistic retreats’. Yogis stressed that the temporary highs from drugs were potentially harmful. True spiritual awakening required extensive training and discipline, while altered states of consciousness were major spiritual experiences not to be treated trivially.^80 81^

Wilson Van Dusen, the Chief Clinical Psychologist at Mendocino State Hospital in Talmage, California—later home to the Buddhist community and monastery The City of Ten Thousand Buddhas—agreed that ‘satori (enlightenment) is better attained by years of meditation’ than by an LSD trip. While Van Dusen ‘would prefer years in a quiet monastery’ to the immediate encounter with the divine afforded by drugs, he admitted that this was not an option for everyone, especially in the USA:

But, in the Western world at least, we don’t have the time. It is curious that a fast approach should come out of Western chemical laboratories! Yet, if LSD is rejected in favor of meditation because the latter is natural and the former not, I can’t agree. In the view of Zen this is a foolish distinction. The Lord designed LSD and LSD experimenters and LSD experience just as much as He designed monasteries and sitting meditation. It does look as though the slower way of Zen meditation lays a more solid foundation, though this needs to be tested too…Personally, if enlightenment could be obtained by sitting in cow dung with ashes on my head, I would do it.^82^

Although critical of some aspects of psychedelic users claiming spiritual growth, Van Dusen could not deny the need for enlightenment in America. Even ‘sitting in cow dung’ was better than nothing.^82^

For right-leaning commentators, and in the run-up to the formal declaration of a War on Drugs, in 1971, the preceding decade had inaugurated an ‘epidemic of drug abuse in the search for means to alter consciousness’. Critics of the counterculture noted that this desire went back millennia and that even ‘yoga, with its transcendental meditation techniques, was an attempt to achieve the psychedelic state’.^83^ Researchers were warned not to be seduced by their appeal: ‘Science is the path we have chosen to aid in man’s growth and development, and mysticism in whatever guise is a contaminant of the scientific attitude’.^84^ Although LSD was initially promoted by Californian intellectuals and those in the psy-sciences in the 1950s, it lost credibility with many in the medical community thereafter.^85^ Nonetheless, it remained firmly entrenched in the counterculture and as part of mental health and spiritual development.

## A breath of fresh air: Grof’s Holotropic Therapy

Special attention must be given to Stanislav Grof. An emigre Czechoslovak psychiatrist, psychedelic researcher and one-time scholar at the Esalen Institute, Grof famously experimented with LSD in 1950s Prague and later in the USA. With an interest in Eastern religion, Grof related how his own psychedelic sessions ‘seemed comparable to the light of supernatural brilliance said in Oriental scriptures’. He went on: ‘The Divine manifested itself and took me over in a modern laboratory in the middle of a scientific experiment conducted in a Communist country with a substance produced in the test tube of a 20th century chemist’. His patients and test subjects regularly reported feelings of transcendence, while he advocated that the brain’s ‘pre-existing matrices’ were activated by psychedelics. But Grof had a hunch that there were other ways of switching this part of the grey matter on.^86^ After moving to California in 1973, he continued to investigate the treatment of mental health conditions by altering patients’ states of consciousness.

When LSD was banned in the late 1960s, Grof developed Holotropic Breathwork with his wife Christina at the Esalen Institute (Grof, p. 255–256).^87 88^ Based on the yogic practice of modulated breathing, the technique used hyperventilation to mimic the ‘experience of a non-ordinary state of consciousness’. Grof contended that his therapy built a patient’s ‘character armour’, strengthening the body and mind. While not as popular as yoga, it developed a loyal following. The technique emphasised non-pharmacological methods of reaching altered states of consciousness as equally important in the treatment of mental illness and ‘spiritual emergencies’. Such emergencies, according to Grof, were psychospiritual crises that might derail an otherwise sound mind (Sessa, p. 63–64; Walsh, p. 219; Grof, p. 256).^4 62 87 89^ ^ix^

In a feature piece for *Yoga Journal* in 1985, Grof recalled how he had mined Eastern thinking to supplement orthodox medical therapies. His goal was to create ‘a bridge between the relatively limited Western point of view and the maps of consciousness provided by the major mystical traditions’. This included Buddhism and yoga. His wife, Christina, discovered the combination when she began practising Hatha Yoga to stay fit during her first marriage. During childbirth, she used the breathing techniques she had honed in class and later argued that ‘this enormous spiritual force was released in me’. The same feeling arose during her second child’s birth. Thereafter, she had ‘spontaneous experiences, all happening within the context of daily life’.^90^ With enough practice, one did not need synthetic substances like LSD or mescaline to achieve altered states of consciousness (Grof, p. 256).^87^ ^x^ Yoga was enough to induce a ‘dramatic inner awakening’.^91^

Christina’s experiences, however, were not wholly positive. At times, she thought she was ‘going crazy’. After she met Grof and he explained psychedelic experiences, Christina realised altered states could help her with her mental health issues. In 1980, the couple founded the Spiritual Emergency Network in California to offer workshops in transpersonal therapy and Holotropic Breathwork to those in crisis. Christina characterised the technique as ‘wiser than we are. But it requires a great deal of trust and surrender’.^92^ It also proved successful against chemical dependency and spiritual crises. Bodywork of this nature allowed her to battle her demons; it enabled her to regain her mental health, and, ‘after years of longing for a felt-contact with the presence I perceive to be God, I am finding that it is possible, with some effort on my part, to feel that connection in every day of my life’.^93^ Synthetic highs might lead to a form of transcendence, but in some renderings this was nothing compared with more naturally induced altered states of consciousness.

## Everything is for sale in America, even bliss

In the early 1990s, the *Yoga Journal* published a series of retrospective articles on the 1960s’ preoccupation with altered states of consciousness. An emphasis on psychedelics was palpable, which reflected the impact such substances had on American yoga culture. Consumers of psychedelics, Eastern religion and yoga in the 1960s were, of course, mostly middle-class to upper-class, white, college-educated liberals; probably, the same people who subscribed to *Yoga Journal* in their later years. As historian David Herzberg has pointed out vis-à-vis tranquillisers and antidepressants, the media played an important role in creating ‘meaning[s] for white middle class culture’.^94^ Messages in *Yoga Journal*, similarly, reinforced ideas about psychedelics and yoga to its target demographic.

One article suggested that the trippers and yogis of the era had simply rediscovered ‘ancient knowledge about the potentials of the human mind’. Americans’ journey deep into Eastern religion was regarded as an attempt to break the ‘consensus trance’ that dominated ‘normal’ life. The meeting of psychedelics and yoga was, according to this view, a natural reaction. ‘We’ve developed in the West a state-limited philosophy and psychology’, the *Yoga Journal* piece contended, “…Buddhist, yogic, Vedantic, Taoist and Sufic philosophies are clearly created from, and designed to induce, multiple states of consciousness. Without experience of those states, we may not be able to appreciate the messages of those other philosophies”.^95^ Still, while the philosophical basis of the counterculture was significant, some saw drug-induced experiences as just as life-changing. In an era where television had ‘made people a lot more stupid’, recalled psychedelic experimenter Starhawk, ‘psychedelics had enormously expanded the range of things that people could think about in this culture’.^96^

Modern yoga practitioners also viewed psychedelics as a shortcut to an altered state of consciousness and superior mental health. Psychedelics offered a sort of inauthentic consciousness of the divine, as articulated by Meher Baba (Osto, p. 264).^8^ ^xi^ Yogis concerned with spiritual purity have not tended to overtly judge drug-takers, but since the 1960s they have regarded psychedelics as an ersatz experience of bliss. Roughly 83% of respondents to a 1996 questionnaire in *Tricycle: The Buddhist Review* admitted to taking psychedelics. Respondents likewise believed they ‘are not a path, but they can provide a glimpse of the reality to which Buddhist practice points’ (Osto, p. 1–2).^8^ Yoga, among other Eastern practices, was marketed to countercultural Americans as an alternative to the false ‘path’ of psychedelics from the mid-1960s. It gave practitioners a ‘safer, more permanent “high”’ than drugs, without the latter’s ‘inherent limitations’.^97^ Without the psychedelic revolution and attendant investigations by mental health practitioners, however, many of those looking for something beyond the America they saw before them would not have discovered non-chemical alternatives like yoga.

This article has traced the contours of psychedelics, yoga and mental health, yet it is clear that there is more work to be done using new sources, approaches and narrower geographical areas. It may have appeared the purists had defeated the proponents of psychedelics, but many yogis and American Buddhists continued to take drugs long after Meher Baba and others had denounced them in the 1960s (Osto, p. 203).^8^ Forces within the countercultural and yoga movements entangled them and it was difficult to parse the two practices. Devotees of altered states of consciousness, inspired by meditation, yoga and Eastern thought, were seen as ‘fringy’ and ‘naïve’. Since the 1960s–1970s, however, the syncretic nature of yoga/psychedelics has conquered the world, with researchers providing data to substantiate the claims made by gurus, yogis and trippers.^98^ In *The Psychedelic Renaissance*, Dr Ben Sessa, a neuroscientist and lifelong student of psychedelic medicine, science and culture, noted that ‘God—if there is such a thing—can, I believe, be known through the psychedelically-induced peak experience’ (Sessa, p. 9).^1^ He is not alone in this estimation.

Yoga was ‘one of the first and most successful products of globalisation, and it has augured a truly post-Christian, spiritually polyglot country’ (Syman, p. 9).^1^ Its adoption by the alternative health and New-Age movements differed from the more institutional syntheses of religion and psychedelics. Yet, it aligned with the historical combination of psychotropic substances and religion, offering up spiritual sustenance, mental health benefits and a more supple body.^99^ ^xii^ The historical record has borne out that the hopes and dreams of psychedelia’s proponents, many of whom were based in California and the East Coast, died at the end of the 1960s. Those that remained in the movement were treated with contempt by the mainstream and portrayed as little more than stoners and burnouts. Once seen as a panacea for the West’s malaise, psychedelics were now believed to be the cause of many of its problems (Sessa, p. 197).^4^

It was, by some accounts, the era ‘of the spiritual wisdom of the unlettered holy man over the intellectual accomplishments of the scholar’.^100^ Yet, mental health professionals clearly impacted how spirituality and altered states of consciousness were scientifically and culturally understood. Psychedelic experiences were ‘literally purchased’ (Grogan, p. 179),^60^ as can yoga classes today.^101^ ^xiii^ Young people rebelled against their parents and society ‘in a kind of war on order and morality’ by taking drugs for ‘a chance to get love and religion at bargain prices’.^102^ Critics like Baba and Maslow lamented the quick and easy pathway to transcendence without having to strive for it as potentially spiritually harmful. ‘I think’, Maslow wrote, ‘it’s clearly better to work for your blessings, instead of to buy them. I think an unearned Paradise becomes worthless’ (Grogan, p. 179).^60^ Despite such warnings, the psychedelic and alternative health movements, however problematic their use of Eastern thought and practices, changed American culture.^103^ Even if the more grandiose designs of the countercultural movement were not achieved, the US grew more open as a result of its experimentation. ‘Some of it is flaky openness, being open to nonsense’, one commentator put it, ‘but a lot of it is a good openness’.^104^ In sum, Americans adapted ancient beliefs and modern drugs to their needs. By combining psychedelics with yoga, they could purchase bliss while going through the motions of spiritual exercise.

## Footnotes

i Up to 90% of cultures have ‘institutionalised forms’ of altered states of consciousness-seeking.

ii Oliver Sacks identified this desire to escape the quotidian and venture into altered states as a basic human impulse and necessity.

iii These were of course explosive times in mental health history. Psychoanalysis, Sigmund Freud’s therapy to unlock the unconscious mind, was struggling against a ‘challenge to the couch’ in a full-scale skirmish with proponents of biological psychiatry. Social psychiatry and deinstitutionalisation movements were reforming traditional mental health services. Community mental health clinics emerged around the country, based on breakthroughs in psychopharmacological research and treatment. Powerful new antipsychotic drugs, that is, enabled transformation in mental health service models.

iv See also the Wellcome Trust funded project The Breath of Life.

v With some notable exceptions, for example, the Pranksters group, who were not at all interested in religion and spread LSD throughout the San Francisco Bay Area.

vi Huxley, ‘Drugs that Shape Men’s Minds’. At other times, he suggested that only intellectuals, writers and artists ought to take these powerful drugs.

vii For a thorough examination of Maslow’s approach to psychedelic spirituality, see David Steindl-Rast, ‘Psychoactive Substances and Sacred Values: Reconsidering Abraham Maslow’s Discoveries’.

viii Similar descriptions of the psychedelic experience can be found in 21st century New-Age publications.

ix The idea that yoga can cure ‘the psychospiritual illnesses of the times’ resonated into the 2010s.

x According to Grof, holotropic states can occur with no obvious trigger and can ‘be induced by powerful forms of experiential therapy’.

xi With the Dalai Lama even contributing to the field of neuroscience.

xii Like Timothy Leary’s community in Millbrook, New York, and the Acid Churches of the 1960s.

xiii For an analysis of the marketisation of Eastern religion in modern America.
